# Editorial: Carbon-water-nitrogen processes and mechanisms of agricultural and forest ecosystems under future climate change

**DOI:** 10.3389/fpls.2024.1423506

**Published:** 2024-05-15

**Authors:** Yunpu Zheng, Qingpeng Yang, Haoran Zhou, Ming Xu

**Affiliations:** ^1^ School of Water Conservancy and Hydropower, Hebei University of Engineering, Handan, Hebei, China; ^2^ Institute of Applied Ecology, Chinese Academy of Sciences (CAS), Shenyang, China; ^3^ School of Earth System Science, Institute of Surface-Earth System Science, Tianjin University, Tianjin, China; ^4^ BNU-HKUST Laboratory for Green Innovation, Advanced Institute of Natural Sciences, Beijing Normal University at Zhuhai, Zhuhai, China; ^5^ Guangdong-Hong Kong Joint Laboratory for Carbon Neutrality, Jiangmen Laboratory of Carbon Science and Technology, Jiangmen, China

**Keywords:** global change, plant growth, carbon sequestration, crop yield, carbon budget, ecosystem structure and function, ecological process models

It is well known that agricultural and forest ecosystems serve as vital carbon sinks in terrestrial ecosystems. Understanding the fundamental processes and mechanisms of ecosystem carbon cycles in face of climate change is critical for quantifying the carbon sinks of terrestrial ecosystems. Ecosystem carbon cycles cannot be separated from water and nitrogen cycles and thus the response and adaptation of carbon-water-nitrogen processes in agricultural and forest ecosystems to climate change demand further studies. This research topic published 10 papers to gain novel insights into the underlying mechanisms and processes of carbon-water-nitrogen interactions in agricultural and forest ecosystems in response to climate change.

Litter decomposition is a pivotal biogeochemical process, which profoundly influences carbon and nitrogen cycling in forest and grassland ecosystems. Climatic factors can significantly impact litter decomposition rates, carbon sequestration, and the emissions of greenhouse gases such as CO_2_ and N_2_O. Liu et al. conducted a comprehensive meta-analysis of 351 samples from 37 published studies to explore the interactive effects of solar radiation and precipitation on litter decomposition and CO_2_ emission on a global scale. They found that the solar radiation significantly increased litter decomposition which was dependent on precipitation regimes. Meanwhile, Li et al. investigated the effects of warming and reclamation on N_2_O emission flux through a long-term manipulative warming experiment on the Qinghai-Tibetan Plateau. Their results demonstrated that reclamation amplified the warming effects on N_2_O emissions by enhancing soil nitrification and related enzymatic activities in alpine meadows. Additionally, Lin et al.examined the long-term spatiotemporal variations in aboveground carbon sequestration rates in the eastern Tibetan Plateau using a forest landscape model under different climate change scenarios. Their study highlighted the variability in aboveground carbon sequestration rates across various forest types in response to climate warming.

The escalating frequency and duration of drought events under climate change have prompted heightened scrutiny of their impacts on temporal and spatial variations in evapotranspiration and water use efficiency. Yang et al. analyzed the inter-annual variations of evapotranspiration with eddy covariance fluxes in an urban forest in Beijing, China, revealing that spring and mid-summer droughts primarily drove inter-annual variations in evapotranspiration due to the reduced stomatal conductance. Furthermore, Xu et al. employed the ensemble empirical mode decomposition method to investigate spatial variations and mechanisms influencing the stability of water use efficiency in China. Their findings explored the role of precipitation and soil moisture in promoting stable water use efficiency, with unstable trends primarily driven by positive or negative reversals. In addition, Jiang et al. presented an in-depth analysis of the impact of seasonal drought on root distribution and water utilization patterns of forests in south China. Their findings highlighted the critical role of deep fine roots in utilizing deep soil water during the dry season, constituting a substantial portion of total water consumption.

Leaf photosynthesis, reliant on both photochemical and carboxylation processes, is central to terrestrial carbon fluxes. Accurate estimation of photochemical parameters and maximum carboxylation rates is essential for predicting carbon fluxes of terrestrial ecosystems. The article by Liu et al. found that the inferred values of the photochemical redox parameters varied with leaf macronutrient contents. The work of Li et al. evaluated the relationships between the leaf maximum rate of carboxylation and both leaf N_area_ and photosynthetic pigments of winter wheat in a farmland ecosystem in China. One of the study’s notable findings was the strongest correlation between leaf *V*_cmax_ and leaf Chl_area_. Their findings suggest that photosynthetic pigment content serves as a predictor for estimating *V*_cmax_, offering a novel approach for spatially continuous *V*_cmax_ estimation and enhancing simulation accuracy in ecological models.

In addition to the above articles from field studies, two papers elucidated the mitigated effects of elevated CO_2_ concentrations on environmental stressors such as drought and nutrient deficiency. Based on manipulative experiment with environmental growth chambers, Chang et al. examined the effects of elevated CO_2_ concentration and temperature on plant growth and leaf gas exchange of winter wheat along a soil water gradient, and found that elevated CO_2_ concentration substantially enhanced leaf photosynthesis by about 30% under water deficiency, suggesting the negative impacts of water deficiency on winter wheat might be partially mitigated by elevated CO_2_ concentration. Li et al.reported findings that the plant growth and leaf photosynthesis of annual ryegrass under phosphorus deficiency were enhanced by elevated CO_2_ concentration, indicating the impacts of phosphorus deficiency on annual ryegrass may be alleviated by elevated CO_2_ concentration under climate change.

This research topic presents the latest studies on how climate change affects the carbon-water-nitrogen processes of agricultural and forest ecosystems. Meanwhile, this research topic highlights the importance and uncertainty of ecosystem functioning in response to climate change. Therefore, fully exploring the potential mechanisms and processes of agriculture and forests to climate change is pivotal to projecting the potential risk of climate change on global grain yield and forest carbon sequestrations.

## Author contributions

YZ: Writing – original draft, Writing – review & editing. QY: Writing – review & editing. HZ: Writing – review & editing. MX: Writing – original draft, Writing – review & editing.

